# The Effectiveness of Web-Based Interventions Delivered to Children and Young People With Neurodevelopmental Disorders: Systematic Review and Meta-Analysis

**DOI:** 10.2196/13478

**Published:** 2019-11-01

**Authors:** Kareem Khan, Charlotte L Hall, E Bethan Davies, Chris Hollis, Cris Glazebrook

**Affiliations:** 1 Division of Psychiatry and Applied Psychology Institute of Mental Health University of Nottingham Nottingham United Kingdom; 2 NIHR MindTech Medtech Co-operative Institute of Mental Health University of Nottingham Nottingham United Kingdom; 3 NIHR Nottingham Biomedical Research Centre Nottingham United Kingdom

**Keywords:** online intervention, effectiveness, neurodevelopmental disorders, children and young people, methodology, systematic review

## Abstract

**Background:**

The prevalence of certain neurodevelopmental disorders, specifically autism spectrum disorder (ASD) and attention deficit hyperactivity disorder (ADHD), has been increasing over the last four decades. Nonpharmacological interventions are available that can improve outcomes and reduce associated symptoms such as anxiety, but these are often difficult to access. Children and young people are using the internet and digital technology at higher rates than any other demographic, but although Web-based interventions have the potential to improve health outcomes in those with long-term conditions, no previous reviews have investigated the effectiveness of Web-based interventions delivered to children and young people with neurodevelopmental disorders.

**Objective:**

This study aimed to review the effectiveness of randomized controlled trials (RCTs) of Web-based interventions delivered to children and young people with neurodevelopmental disorders.

**Methods:**

Six databases and one trial register were searched in August and September 2018. RCTs were included if they were published in a peer-reviewed journal. Interventions were included if they (1) aimed to improve the diagnostic symptomology of the targeted neurodevelopmental disorder or associated psychological symptoms as measured by a valid and reliable outcome measure; (2) were delivered on the Web; (3) targeted a youth population (aged ≤18 years or reported a mean age of ≤18 years) with a diagnosis or suspected diagnosis of a neurodevelopmental disorder. Methodological quality was rated using the Joanna Briggs Institute Critical Appraisal Checklist for RCTs.

**Results:**

Of 5140 studies retrieved, 10 fulfilled the inclusion criteria. Half of the interventions were delivered to children and young people with ASDs with the other five targeting ADHD, tic disorder, dyscalculia, and specific learning disorder. In total, 6 of the 10 trials found that a Web-based intervention was effective in improving condition-specific outcomes or reducing comorbid psychological symptoms in children and young people. The 4 trials that failed to find an effect were all delivered by apps. The meta-analysis was conducted on five of the trials and did not show a significant effect, with a high level of heterogeneity detected (n=182 [33.4%, 182/545], 5 RCTs; pooled standardized mean difference=–0.39; 95% CI –0.98 to 0.20; Z=–1.29; *P*=.19 [I^2^=72%; *P*=.006]).

**Conclusions:**

Web-based interventions can be effective in reducing symptoms in children and young people with neurodevelopmental disorders; however, caution should be taken when interpreting these findings owing to methodological limitations, the minimal number of papers retrieved, and small samples of included studies. Overall, the number of studies was small and mainly limited to ASD, thus restricting the generalizability of the findings.

**Trial Registration:**

PROSPERO International Prospective Register of Systematic Reviews: CRD42018108824; http://www.crd.york.ac.uk/PROSPERO/display_record.php?ID=CRD42018108824

## Introduction

### Background

Web-based interventions for children and young people (CYP) with physical and psychological problems are relatively new phenomena, with the first trials of internet-delivered therapies being conducted in the late 1990s [1]. However, they are very important developments in the access to health care and treatment for CYP with long-term chronic health conditions. Neurodevelopmental disorders (NDDs) are a group of disorders that typically manifest early in development and are characterized by deficits in cognitive function, motor function, verbal communication, social skills, and behaviors [2]. Common NDDs include autism spectrum disorder (ASD), attention deficit hyperactivity disorder (ADHD), specific learning disorder (including dyscalculia and dyslexia), intellectual disability (ID), and tic disorder ([TD], including tourette syndrome and chronic tic disorder [CTD]) [3]. NDDs frequently co-occur, for example, individuals with ASD often have ID, and many children with ADHD have a specific learning disorder [3]. CYP with NDDs also have complex comorbidities and related symptoms, such as depression and anxiety [4]. There is growing evidence that the impact of NDD is lifelong for many individuals [5], and although exact prevalence rates of NDDs vary considerably between countries, researchers suggest that the prevalence of certain NDDs, specifically ASD and ADHD, has been increasing over the last four decades [6-8].

Psychological therapeutic interventions exist for a range of NDDs. These include therapies to manage NDD symptoms, such as habit reversal therapy for TDs, behavioral therapy to alleviate commonly associated symptoms, such as cognitive behavioral therapy (CBT) for anxiety symptoms, and psychoeducation to facilitate the management of NDDs. Owing to their complexity and chronic nature, pharmacotherapy may often be used as part of a treatment plan [9]. However, pharmacological interventions are considered undesirable for children because of the associated side effects [10]; therefore, psychological treatment is more desirable. A major barrier to psychological treatment is difficulty in accessing appropriately trained therapists, because of the limited numbers of therapists in child mental health services relative to the demand and the uneven geographical distribution of services. It is likely that Web-based therapy can help increase the availability and uptake of evidence-based interventions, offering the opportunity to deliver less therapist-intensive but effective interventions over long distances. Given that Web-based technology is a ubiquitous part of everyday life and young people are by far the highest users [11], Web-delivered therapy is intuitively attractive for CYP.

Web-based interventions are self-guided or therapist-assisted programs with the aim of improving knowledge, providing support, care, or treatment to a diverse population with a range of health problems. In the field of psychological and neurodevelopmental health, Web-based therapeutic interventions have been designed for CYP with a range of problems including ADHD [12], anxiety [13], depression [14], and obsessive-compulsive disorder [15]. These interventions all differ in the type of therapy delivered, their level of participant interaction with the program, number of sessions (dosage), level of trained expert support, structure, modality, and whether there is a parent component or not. However, little is known about what characteristics are integral to efficacious Web-based interventions, especially for CYP. There is some literature in adult populations to suggest that guided Web-based interventions are more efficacious than self-guided or unguided interventions [16], and the most effective interventions tend to be individualized to the user and more intensive [17]. To improve the future developments of Web-based interventions, it would be beneficial to synthesize the evidence for characteristics of effective interventions in CYP to minimize the risk of developing inadequate and ineffective interventions.

A preliminary search conducted in PROSPERO, the Cochrane Database of Systematic Reviews, and the Joanna Briggs Institute (JBI) Database of Systematic Reviews and Implementation Reports indicated that there are no systematic reviews in progress or already published on CYP with NDDs.

### Objectives

The objective of this review was to evaluate the effectiveness of Web-based interventions for CYP with NDDs and conduct a meta-analysis of the most effective intervention characteristics (eg, therapist-supported vs stand-alone) with the aim of informing the future development of technologies. The findings will also be useful to health care providers, commissioners, and clinicians in informing future clinical developments in the delivery of care.

## Methods

The systematic review was registered on PROSPERO (registration number: CRD42018108824) and conducted in accordance with the JBI methodology for systematic reviews of effectiveness evidence.

### Search Strategy

An initial limited scoping search of Medical Literature Analysis and Retrieval System Online (MEDLINE) was undertaken to identify relevant articles. The text words contained in the titles and abstracts of relevant articles and the index and Medical Subject Headings terms describing the articles were used to develop a full search strategy, which was then tailored for each included information source (see Multimedia Appendix 1 for full search strategy). Search terms were related to NDDs, Web-based interventions, and adolescence.

A total of 6 electronic databases—including PsycINFO, PubMed, EMBASE, Cochrane Central Register of Controlled Trials, Web of Science, and MEDLINE—were searched in August and September 2018. One trial register (ClinicalTrials.gov) was also searched. The reference list of all studies selected for critical appraisal was screened for additional studies, and several specialized journals, publisher websites, and published reviews were hand-searched. As Web-based interventions are a recent development and older interventions will now be obsolete, the year of publication was limited from 2000 to September 5, 2018. There were no restrictions on the language of publication.

Studies were included if they met the following criteria:

1. The intervention aimed to improve the diagnostic symptomology of the targeted NDD as measured by a valid and reliable outcome measure.
2. The intervention was delivered on the Web via a website, a mobile app, social media, an email, or a personal digital assistant. The intervention could include human support in its delivery.
3. The study was an RCT design and published in a peer-reviewed journal. Trial arms needed to consist of an experimental group compared with no treatment and/or another active intervention or treatment as usual (TAU) or waitlist control.
4. The intervention was targeted at a youth population (aged ≤18 years or reported a mean age of ≤18 years) with a diagnosis or suspected diagnosis of the following NDDs: communication disorders (eg, language disorder and stuttering); ASD; ADHD; specific learning disorder (eg, dyslexia and dyscalculia); motor disorders; TD; other NDDs (eg, NDD associated with prenatal alcohol exposure).

These disorders were selected based on the Diagnostic and Statistical Manual of Mental Disorders, Fifth Edition (DSM-5) criteria [3].

Secondary outcomes of interest were comorbid or associated psychological symptomology and any adverse events. Papers had to report on either primary or secondary outcomes of interest to be included in this review. Studies were excluded if the intervention was not delivered on the Web or was primarily aimed at the parent or caregiver. Furthermore, we excluded studies where the participants were diagnosed with IDs as intervention characteristics that meet the needs of children with significant IDs would be difficult to generalize to a youth population as a whole. Moreover, studies on NDDs frequently exclude CYP with any form of learning difficulty because of their unique complexity [18].

**Figure 1:**
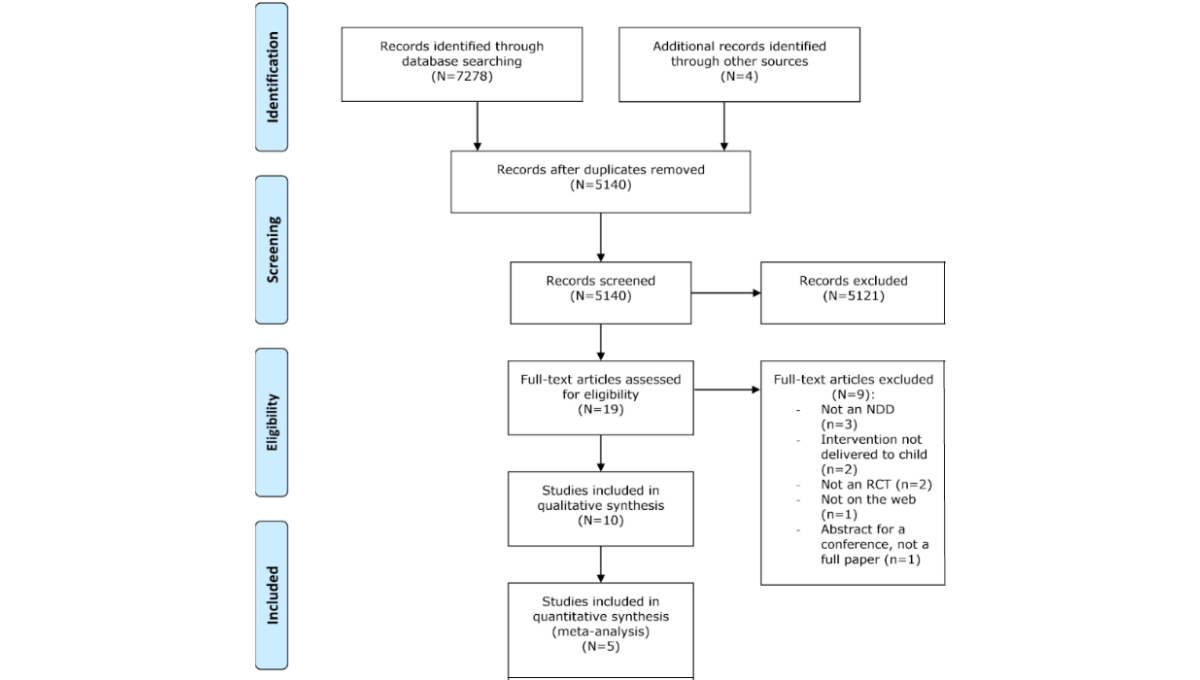
Preferred reporting items for systematic reviews and meta-analyses flowchart outlining the process for systematic review and meta-analysis. NDD: neurodevelopmental disorder; RCT: randomized controlled trial.

Once duplicates were removed (n=2142), a total of 5140 titles and abstracts were retrieved. Titles were initially screened against the eligibility criteria by 1 assessor (screening phase, n=4985 ineligible). Subsequently, 155 titles and abstracts were then screened against the eligibility criteria by 2 independent assessors. Any conflicts concerning eligibility were resolved by group discussion. There was agreement on 7 papers to be included, 121 to be excluded, and 27 papers requiring further discussion. Following a discussion between the assessors, the full text of 19 papers was obtained for further analysis and coding. A consensus was reached among the assessors on 9 papers to be excluded, as they did not meet the eligibility criteria, leaving 10 papers for analysis. Figure 1 shows the Preferred Reporting Items for Systematic Reviews and Meta-analyses flowchart [19].

### Data Extraction

The first assessor extracted the following data from all included studies: specific details about the study (authors, year, number of study arms, location, and Web-based program name), population demographics (sample size, age, and gender), study methods, interventions and comparisons, length of treatment or dosage, condition treated (eg, ASD and ADHD), outcome measures, type of analysis (eg, intention-to-treat [ITT]), and primary and secondary outcomes of significance to the review. These data were extracted and inputted into JBI System for the Unified Management, Assessment and Review of Information (SUMARI) software [20]. Missing data were obtained from the manuscripts, and where these data were not documented, the primary authors were contacted for relevant information.

### Assessment of Methodological Quality

A total of 2 independent assessors examined the methodological quality of included studies using the JBI RCT appraisal tool in JBI SUMARI [20]. Further details on the assessment of quality are provided in Multimedia Appendix 2.

### Meta-Analysis

Continuous variables were examined using standardized mean differences (SMD) with 95% confidence intervals. Extracted continuous data were tested for normality using skew plots. Random effects meta-analyses were performed to compute overall estimates of treatment outcomes. The effect sizes of the primary studies were presented in a forest plot. Heterogeneity was examined with the I^2^ statistic [21]. The I^2^ statistic calculates the degree to which there is heterogeneity, with 25% suggesting low heterogeneity, 50% indicating moderate, and 75% indicating the threshold for high heterogeneity. The *Q* statistic was also calculated and provides the statistical significance (*P* value <.05) of heterogeneity.

In the protocol, subgroup analyses were planned to be conducted according to the main intervention characteristics that were shown to be the most effective, for example, therapist support versus no support and parent component versus no parent component. However, because of the low number of included studies in the review, this was deemed unsuitable and is therefore a deviation from the protocol. All data for the meta-analysis were conducted using JBI SUMARI [20].

## Results

### Study Characteristics

The search generated 10 studies. A total of 5 interventions targeted ASD [22-26], 2 were aimed at CYP with TD [27,28], 1 for ADHD [29], 1 for specific learning disorder (LD) with poor visual-motor integration (VMI) [30], and the other targeting dyscalculia [31]. All but one of the interventions focused on treating the primary diagnosis with the other focusing on treating comorbid anxiety [22]. All studies used the standard RCT design, except for one study, which employed a crossover RCT design [29].

In 5 studies, NDD diagnosis was confirmed by DSM-IV or DSM-5 criteria [23,25-27,29] with the other studies using disorder-specific diagnostic tools [22,24,30,31]. All 10 studies contained 2 trial arms with the intervention being compared with another active intervention, which was not Web-based [27,30,31], TAU, which was either standard therapy or participants were not prevented from using therapy; however they were told not to use any apps designed for ASD therapeutic use [23,26,29] or waitlist control [22,24,25,28]. A summary of the characteristics of each study is shown in Table 1.

**Table 1:** Characteristics of included studies.

Study	Design, number of arms (N per arm), sample size and study location	Sample demographics and condition treated	Control or comparator group	Outcome measures	Summary of main findings or effect of intervention
Conaughton et al, 2017 [22]	Randomized controlled trial (RCT) 2 arms: Intervention=21, control group=21, N=42, Australia	Children (8-12 years; mean 9.74; 85.7% male) with high-functioning autism spectrum disorder and an anxiety disorder	Waitlist control (WLC)	Anxiety Disorders Interview Schedule: parent and child, Children’s Global Assessment Scale, Child Behaviour Checklist, Spence Children’s Anxiety Scale–child, satisfaction with treatment	9.5% of the intervention group versus 0% of the WLC group had lost all anxiety diagnoses at postassessment, with 14.3% of the intervention group being free of all anxiety diagnoses at 3-month follow-up; the intervention had a positive effect
Esposito et al, 2017 [23]	RCT 2 arms: Intervention=15, control group=15, N=30, Europe	Children (2-5 years; mean 3.92; 90% male) with Autism Spectrum Disorder (ASD) who followed face-to-face (F2F) applied behavior analysis (ABA) treatment	Treatment as usual (TAU)	Measured attention, imitation of actions with objects, receptive identification of objects	Intervention group, who had daily practice of attention and identification of objects on tablet apps, showed greater progress within standard ABA therapy than the TAU group for all 3 programs investigated; however, this did not exceed the significance level (all *P* values >.05); the intervention had no effect
Fletcher-Watson et al, 2016 [24]	RCT 2 arms: Intervention=27, control group=27, N=54, Europe	Children (<6 years; mean 4.13; 79.6% male) with ASD	WLC	The Autism Diagnostic Observation Schedule, Brief observation of social communication change, MacArthur Communicative Development Inventory (MCDI), Communication and Symbolic Behaviour Scales–Developmental Profile, parent impressions of the app	Change scores on all outcome measures revealed no significant differences between intervention and WLC groups (all *P* values >.05); the intervention had no effect
Fridenson-Hayo et al, 2017 [25]	RCT 2 arms: Intervention=43, control group=40, N=83, Europe	Children (6-9 years; mean 7.29; 79.5% male) with ASD	WLC	Emotion recognition (ER) tasks, Wechsler Intelligence Scale for Children or Wechsler Primary and Preschool Scale of Intelligence, Social Responsiveness Scale, Vineland Adaptive Behaviour Scales (VABS-II)	Pairwise comparisons for the time by group interaction revealed that significant improvement over time was found on all ER tasks for the intervention group but not for the WLC group; the intervention had a positive effect
Whitehouse et al, 2017 [26]	RCT 2 arms: Intervention=41), control group=39, N=80, Australia	Children (<4 years; mean 3.32; 78.7% male) with ASD	TAU	The Autism Treatment Evaluation Checklist (ATEC), The Mullen Scales of Early Learning, VABS-II, MCDI, Communication and Symbolic Behaviour Scales, Repetitive Behaviour Scale-Revised , Behaviour Flexibility Rating Scale	No significant differences were observed between groups for any of the 4 ATEC subscales at either the 3- or 6-month assessments, although the 3-month communication subscale showed a trend toward greater improvement in the intervention group, 2.1 units (95% CI 4.5 to 0.3; *P*=.08); the intervention had no effect
Himle et al, 2012 [27]	RCT 2 arms: Intervention=10, comparator group=10, N=20, North America	Children (8-17 years, mean 11.6, 94% male) with tic disorders (TD) or chronic tic disorders (CTD)	F2F Comprehensive Behavioural Intervention for Tics	Yale Global Tic Severity Scale (YGTSS), Clinical Global Impression-Improvement Scale (CGI-I), Parent Tic Questionnaire (PTQ), Treatment Acceptability Questionnaire (TAQ)	The videoconferencing group showed a mean YGTSS reduction of 6.4 points versus 4.2 points for the F2F group at follow-up; both interventions were effective in reducing tics however, there was a slightly better effect on the intervention group at both post-treatment and follow-up compared with the F2F group
Ricketts et al, 2016 [28]	RCT 2 arms: Intervention=12, control group=8, N=20, North America	Children (8-16 years; mean 12.16; 64.9% male) with TD or CTD	WLC	YGTSS, CGI-I, PTQ, Children’s Perception of Therapeutic Relationship, Client Satisfaction Questionnaire, TAQ, Videoconferencing Satisfaction Questionnaire	In the intervention group, there was a statistically significant decrease of 7.25 points in YGTSS total scores from baseline to postassessment. In the WLC group, the 1.75-point decrease on the YGTSS total scores from baseline to postassessment was not significant; the intervention had a positive effect
Bul et al, 2016 [29]	Crossover RCT 2 arms: Intervention=88, comparator group=82, N=170, Europe	Children (8-12 years; mean 9.85; 80.6% male) with attention deficit hyperactivity disorder	TAU crossover group	Time management questionnaire, Behaviour Rating Inventory of Executive Function (subscale plan or organize), Social Skills Rating System (subscale cooperation), It’s About Time Questionnaire, self-efficacy, satisfaction	Intervention group achieved significantly greater improvements on the primary outcome of time management skills compared with TAU crossover group (parent-reported; *P*=.004) and on secondary outcomes of responsibility (parent-reported; *P*=.04), and working memory (parent-reported; *P*=.02); the intervention had a positive effect
Coutinho et al, 2017 [30]	RCT 2 arms: Intervention=10, comparator group=10, N=20, North America	School-aged children (4-7 years; mean 6.18; 12 males) with a specific learning disorder such as dyspraxia or speech delay with poor visual-motor integration (VMI) skills	Traditional occupational therapy sessions	Beery VMI, Miller function and participation scales, intervention appreciation scale	There were some improvements in VMI skills in both groups; however, the finding was not statistically significant; the intervention had no effect
De Castro et al, 2014 [31]	RCT 2 arms: Intervention=13, control group=13, N=26, South America	Primary school children (7-10 years; mean 8.11; 16 male) with dyscalculia	Traditional teaching techniques	Scholastic Performance Test	The intervention using the virtual environment yielded a significant score improvement (*P*<.001) with an average score improvement of 5.09 posttest, whereas the control group did not show a statistically significant score improvement (*P*=.05); the intervention had a positive effect

### Modality, Location, and Duration of Intervention

A total of 4 interventions were delivered via apps [23,24,26,30], 2 were serious games [25,29], 2 used videoconferencing [27,28], 1 was a virtual environment with playable games [31], and the other was a Web-based CBT intervention [22]. Most of the interventions were accessed from participants’ own homes, except 3 studies where participants were based in a rehabilitation center [30], school [31], and hospital or clinic setting [27]. Interventions either had a varying range of components (ie, tasks to be completed)—2 [24,29], 3 [23], and 4 [25,26] components—or sessions, ranging from 8 [27,28] to 10 [22,30,31] sessions. All trials instructed participants on an optimum length of time to access the intervention: ranging from 5 min per day or 10 min every other day [24], 20 min daily [26] and 30 min per day [23] to approximately 2 hours per week [25], one 60-min session per week [22], 2 40-min sessions per week [30], 60 min twice per week [31], and 65 min 3 times per week [29]. The 2 trials comparing Web-based comprehensive behavioral intervention for tics (CBIT) stated that participants received 6 weekly sessions followed by 2 biweekly sessions [27] and 2 1.5-hour sessions followed by 6 1-hour sessions [28]. The intervention delivery period ranged from 4 [23] to 24 weeks [26], with a median length of 10 weeks.

A summary of the characteristics of each intervention is shown in Table 2.

**Table 2:** Characteristics of interventions

Study	Intervention, modality, and aim of the intervention	Length or dosage, follow-ups	Therapist supported	Parent component
Conaughton et al [22]	Internet trans diagnostic CBT^a^ intervention aimed at improving comorbid anxiety symptoms	10 weeks, 10 sessions—one 60-min session per week	Yes	Yes
Esposito et al [23]	Tablet apps aimed at improving attention and identification of objects	4 weeks, 3 app components—30 min daily	Yes	Yes
Fletcher-Watson [24]	iPad app aimed to improve social communication skills	2-months, 2 parts–5 min per day, or 10 min every other day	No	No
Fridenson-Hayo et al [25]	An internet-based serious game aimed at improving emotion recognition	8-12 weeks, 4 components—2 hours per week	No	Yes
Whitehouse et al [26]	iPad app aimed at improving developmental skills relevant to autism	6 months, 4 components–20 min per day	No	Yes
Himle et al [27]	Internet-accessed videoconference aimed at improving tic severity	10 weeks—6 weekly sessions followed by 2 biweekly sessions	Yes	Yes
Ricketts et al [28]	Internet-accessed videoconference (Skype) aimed at improving tic severity	10 weeks—2 1.5-hour sessions followed by 6 1-hour sessions	Yes	Yes
Bul et al [29]	An internet-based serious game aimed at improving time management and planning skills	10 weeks, 2 game components—65 min approximately 3 times per week	No	No
Coutinho et al [30]	Multiple iPad apps aimed at improving visual motor skills	10 weeks, minimum of 8 and maximum of 12 sessions—2 40-min sessions per week	No	No
De Castro et al [31]	Internet-accessed virtual environment aimed at improving mathematical skills	5 weeks, 10 sessions—60 min twice a week	No	No

^a^CBT: cognitive behavioral therapy.

### Use of Human and Technical Support

In total, 4 interventions were therapist assisted [22,23,27,28]; however, all these differed in the level of involvement of the therapist within the interventions. The contacts ranged from once weekly contact [22] 2 hours per week [23], and the 2 trials of CBIT were exclusively therapist-delivered [27,28].

One of the major factors that developers need to consider when creating Web-based intervention is the ease with which nontechnologically advanced individuals can access and use the program. Thus, it is crucial to provide technical support as and when needed. In total, 7 of the 10 included studies reported the use of technical support. In 2 trials [22,28], participants had weekly access to a therapist who was able to offer any technical assistance within the sessions. One trial [30] took place within a rehabilitation center with an occupational therapist (OT) constantly present to offer any assistance. Two trials reported the use of monitoring phone calls from research personnel to check for any issues, which were offered either fortnightly [26] or once a week [25]. In both of these trials, parents were also encouraged to contact research staff with any queries or issues in between monitoring calls. In one trial [27], research personnel were available to manage any technical difficulties. In the other trial [23], parents were fully trained in the apps by research staff and were taught how to handle technical difficulties.

### Participant Characteristics

A total of 545 participants consented and were randomized to a trial arm. Sample sizes ranged from 20 [27,28,30] to 170 [29] participants. A total of 4 trials had sample sizes of >50 participants [24-26,29]. Overall, 523 participants were explicitly included in analyses. A total of 5 studies stated that the analysis was conducted on participants who completed pre- and postintervention measures only [23,25,27,30,31], whereas 5 conducted ITT analyses [22,24,26,28,29]. All 10 trials reported participant dropout or withdrawal data, with dropout rates ranging from 0% [23,28,31] to 18% (n=31) of the sample [29]. Reasons for participant withdrawal included lack of motivation or disinterest [25,29], lack of enjoyment with the intervention [24,26], and personal reasons [26].

In the 10 trials, participants ranged in age from 2 to 17 years, with a mean age ranging from 3.32 to 12.16 years. Males were the majority in all studies, with gender balance varying from 62.5% [31] to 94% [27] of the sample being male. A total of 4 trials were conducted in Europe [23-25,29], 3 in North America [27,28,30], 2 in Australia [22,26], and 1 in South America [31].

### Provider Characteristics

Most of the trials recruited participants from clinics [22-25,29], with 3 studies [25,26,28] recruiting via advertisements and 1 study [28] recruiting participants through solicitations mailed to health care professionals. One study [26] recruited participants through referrals from diagnosing clinicians, and another study [22] utilized referrals through general practitioners, mental health professionals, school guidance officers, teachers, parents, and media publicity.

### Adverse Events and Outcome Measures

Only 1 study [29] explicitly stated that they recorded and reported adverse events. The crossover trial investigating the effects of a serious game as an adjunct to TAU for children with ADHD reported 10 adverse events in the trial that could be related to the intervention, and parents, teachers, or participants themselves reported these. Adverse events were registered as mild (n=5) or moderate (n=5) in severity and examples included pain in the fingers, irritability, and headache. One participant could not concentrate at school and therefore discontinued from the trial because of this adverse event; however, no serious adverse events were reported.

It was estimated that the outcome measurement battery ranged from 16 [24] to 175 items [26] at each time point of the studies. The estimated median number of questions administered to participants was 56 items (see Multimedia Appendix 2 for more details).

### Methodological Quality and Risk of Bias

The JBI Critical Appraisal Checklist for RCTs provided a framework for scoring the quality of the included studies by addressing different aspects of the research such as randomization, allocation concealment, blinding, and follow-up data. The methodological quality of included studies was felt to be moderate, mostly because of trials providing insufficient details or being unclear in their reporting (see Table 3). Only 5 of 10 studies reported their randomization methodology [22,24,28-30]. Blinding was the main issue of quality in included studies. A total of 6 trials stated that participants were not blind to treatment assignment with the other 4 trials being unclear in their reporting. Only 1 study [23] reported that those delivering treatments were blind to treatment assignment with the others stating researchers delivering treatment were either not blinded or it was unclear. Half of the trials [22-24,26,27] reported outcome assessors were blind to treatment assignment with all of these studies employing independent researchers to carry out assessments.

**Table 3:** Critical appraisal of included studies.

Study	Q1^a^	Q2^b^	Q3^c^	Q4^d^	Q5^e^	Q6^f^	Q7^g^	Q8^h^	Q9^i^	Q10^j^	Q11^k^	Q12^l^	Q13^m^
Conaughton et al [22]	Yes	Yes	Yes	Unclear	No	Yes	No	Yes	Yes	No	Yes	Yes	Yes
Esposito et al [23]	Unclear	Unclear	Yes	Unclear	Yes	Yes	Yes	Yes	Yes	Yes	Yes	Yes	Yes
Fletcher-Watson et al [24]	Yes	Yes	Yes	No	Unclear	Yes	Yes	Yes	Yes	Yes	Yes	Yes	Yes
Fridenson-Hayo et al [25]	Unclear	Unclear	Yes	No	No	Unclear	Yes	Yes	Unclear	Yes	Yes	Yes	Yes
Whitehouse et al [26]	Unclear	Unclear	Yes	No	No	Yes	Yes	Yes	Yes	Yes	Yes	Yes	Yes
Himle et al [27]	Unclear	Unclear	Yes	No	No	Yes	Yes	Yes	No	Yes	Yes	Yes	Yes
Ricketts et al [28]	Yes	Unclear	Yes	No	No	Unclear	Yes	Yes	Yes	Yes	Yes	Yes	Yes
Bul et al [29]	Yes	No	Yes	No	Unclear	Unclear	Yes	Yes	Yes	Yes	Yes	Yes	Yes
Coutinho et al [30]	Yes	Unclear	Yes	Unclear	Unclear	Unclear	Yes	Yes	Yes	Yes	Yes	Yes	Yes
De Castro et al [31]	Unclear	Unclear	Yes	Unclear	Unclear	Unclear	Yes	Yes	Unclear	Yes	Yes	Yes	Yes
Number that met the criteria (%)	50	20	100	0	10	50	90	100	70	90	100	100	100

^a^Q1: True randomization.

^b^Q2: Allocation concealed.

^c^Q3: Treatment groups similar at the baseline.

^d^Q4: Participants blind to treatment.

^e^Q5: Those delivering intervention blind to treatment.

^f^Q6: Outcome assessors blind to treatment.

^g^Q7: Treatment groups treated identically.

^h^Q8: Follow-up complete and if not, differences between groups adequately described and analyzed.

^i^Q9: Participants analyzed in the groups to which they were randomized.

^j^Q10: Outcomes measured in the same way for groups.

^k^Q11: Outcomes measured reliably.

^l^Q12: Appropriate statistical analysis.

^m^Q13: Appropriateness of trial design and any deviations from RCT design accounted for.

### Effectiveness of Web-Based Interventions

Of 10 trials, 6 trials found that Web-based interventions were effective in reducing NDDs or associated symptoms in CYP [22,25,27-29,31]; 2 were serious games, 2 were delivered by videoconferencing, 1 was a virtual environment, and the other was an internet-delivered CBT intervention. Targeted NDD conditions of the effective interventions included ASD [22,25], TD [27,28], ADHD [29], and dyscalculia [31]. All but 2 of the effective interventions were delivered over a period of 10 weeks, and these 2 were delivered over 5 weeks with 10 sessions [31] and 8 to 12 weeks with 4 components [25]. The 4 trials, which did not find that Web-based interventions had an effect on NDD symptoms, were all delivered by apps [23,24,26,30]. All but one of these was designed for CYP with ASD, the other being designed for specific LD with VMI [30].

### Primary Outcomes

Of 10 interventions, 4 interventions in the included studies were aimed at a youth population with ASD; however, just one [25] of these trials found that Web-based interventions were effective. In the study by Fridenson-Hayo et al [25], children with ASD who received an internet-based serious game improved in ER tasks compared with the WLC group who received TAU. A total of 3 studies [23,24,26] comparing iPad or tablet apps with WLC/TAU groups for children with ASD found no difference in outcome between the groups.

Both studies evaluating the effectiveness of internet-delivered CBIT via videoconferencing for young people with TD/CTD showed it could be effective for reducing tic symptomology. Overall, the studies were of similar design but used different comparators with Himle et al [27] using F2F CBIT in their study whereas WLC was utilized in a study by Ricketts et al [28]. The YGTSS was the main primary measure in both trials.

There were 3 other studies that looked to improve primary symptoms in CYP, and these were targeted at CYP with NDDs other than ASD or TD. One study showed improvements in time management skills for children with ADHD [29], and another study found improvements in mathematical skills for children with dyscalculia [31]. The other study found no effect in VMI scores [30]. Secondary outcomes are discussed in Multimedia Appendix 2.

### Satisfaction or Acceptability of the Intervention

A total of 4 trials included participant satisfaction measures [22,24,26,29] and 2 trials administered participant acceptability questionnaires [27,28]. In the study by Bul et al [29], both children and parents reported moderate to high satisfaction with receiving the serious game intervention. In the study by Conaughton et al [22], children and parents reported moderate levels of satisfaction following treatment. In the study by Fletcher-Watson et al [24], parents gave verbal comments on the app and what they perceived to be their child’s response to it. Replies were categorized as *Positive*, *Mixed,* or *Negative*, and there were positive responses to questions on overall experience with the app, whether the child and parent liked the app, and ease of use. In the other study to measure participant satisfaction [26], caregivers of children in the Therapy Outcomes By You (TOBY) intervention group were asked to list up to 3 features that they liked or disliked about the app. The most frequent *like* statement related to TOBY providing a helpful therapy-planning tool. Other common statements were that TOBY was easy to use and that the app provided a positive learning experience for their child with an attractive structure and layout. The most common *dislike* statement was that the offline iPad activities were too time-consuming to prepare. The 2 trials evaluating VC administered CBIT [27,28] gathered acceptability ratings from participants. In both studies, children and parents gave high acceptability ratings for the intervention.

### Meta-Analysis

**Figure 2:**
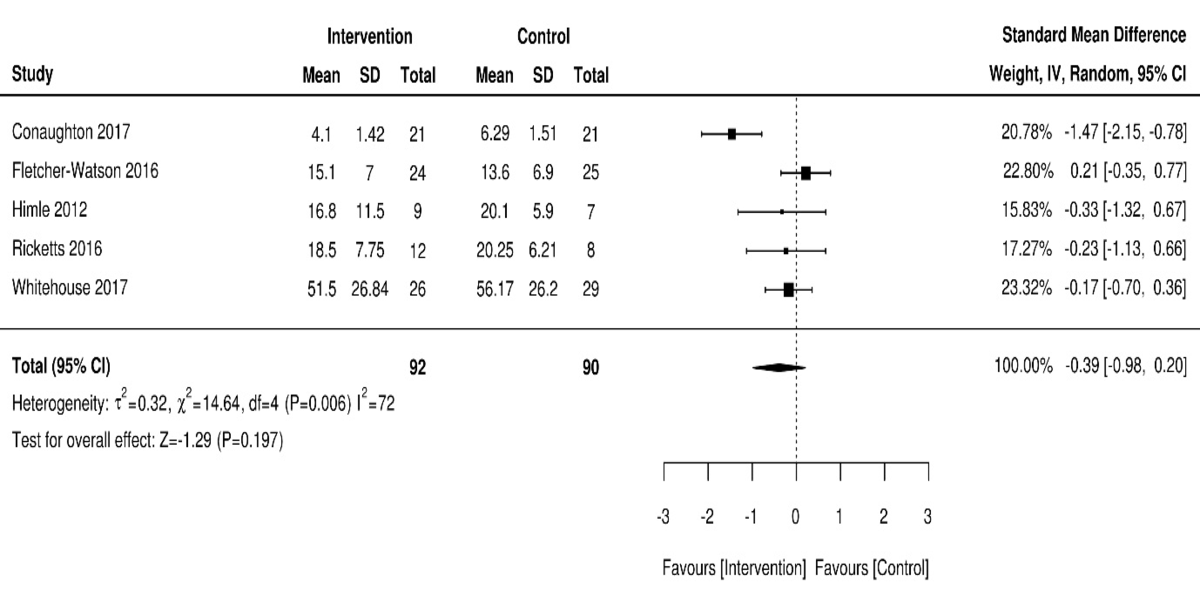
Forest plot of postintervention neurodevelopmental disorder outcomes for intervention compared with controls.

In studies that used a valid and reliable outcome measurement of NDD and associated symptoms, a meta-analysis was undertaken. All outcomes were continuous and scale-based and were extracted as endpoint average scores with lower scores indicating less severe symptomology. The outcomes combined for the meta-analysis were anxiety [22], social communication [24], developmental skills [26], and tic severity [27,28]. Negative SMD values support the intervention in the presented analyses. Figure 2 shows the forest plot for the data.

A total of 5 trials investigated the effects of Web-based interventions on NDD symptoms using a valid, standardized outcome measure to explore symptom reduction. Within the 5 trials, neither intervention nor control was favored, with a high level of heterogeneity detected: 182/545 (33.4%), 5 RCTs, pooled SMD=–0.39; 95% CI –0.98 to 0.20; Z=–1.29; *P*=.19 (I^2^=72%; *P*=.006).

## Discussion

### Principal Findings

We set out to evaluate whether RCT evidence showed Web-based interventions were effective for CYP with NDDs and/or associated symptoms. Our review retrieved 10 studies in total. A further meta-analysis was conducted on 5 of the 10 studies. Most of the interventions targeted ASD in CYP. Overall, the meta-analysis indicated no difference between the intervention and control groups; however, with 6 of the 10 retrieved papers showing a positive effect, the findings suggest that Web-based interventions can be effective in reducing NDD symptoms in CYP. However, the evidence is inconclusive owing to the limited number of retrieved studies and small sample sizes in included trials. The findings indicate the need for further research in the use of Web-based interventions aimed at CYP with NDDs.

Furthermore, one of our initial aims was to evaluate the main characteristics of effective Web-based interventions. A parent component as an adjunct to the main intervention was utilized in 4 of the 6 effective trials, indicating the potential importance of assisted interventions and in line with previous research [16,32,33]. Having a parent component within the interventions is unsurprising given the young age of participants in the included studies. It is more likely that younger children will require some form of parental assistance with digitized interventions and, more generally, therapeutic interventions. Indeed, Thirwall et al [34] found that younger children showed a greater improvement in anxiety symptoms having received a parent-delivered CBT intervention. From this review, it is unclear whether a therapist-supported Web-based intervention is more efficacious than one without, as only half of the effective interventions were therapist supported. Another important characteristic to consider is the length of the intervention. A total of 5 of the 6 effective interventions were delivered over a period of 10 to 12 weeks, with the other having 10 sessions delivered over 5 weeks. This suggests that 10 to 12 weeks/sessions is the optimum length for a Web-based intervention. However, given the high heterogeneity between the Web-based interventions and number of multifaceted aspects to these interventions in this review, caution should be taken when trying to establish certain characteristics that may be relevant in determining effectiveness.

All 4 of the included interventions delivered by apps were unsuccessful in yielding statistically significant outcomes. This suggests apps may not be a promising platform for delivering therapeutic interventions, at least to CYP with NDDs. Indeed, recent systematic reviews [35,36], have shown there is inconclusive evidence on the efficacy of mobile apps utilized as health interventions, despite the high user acceptability ratings of smartphone apps. One interpretation of this finding is that because apps are a new phenomenon—the first mobile apps being developed in 2008 with the advent of Apple’s App Store [37]—little is known about their mechanisms of impact, especially in the health care domain. There are over 10,000 mental health apps commercially available [38], with 52% of smartphone owners using their phones for health purposes and 19% using health apps [39], it is clear that more high-quality research needs to be conducted. As 3 of the 4 apps that found no effect were targeted at CYP with ASD, another interpretation of this finding could be that apps are an insufficient modality for producing positive outcomes in autism-related disorders. This corroborates the results of a study conducted by Grynszpan et al [40]. They found that adolescents with ASD performed poorly on rich multimedia interfaces, such as apps, as they lacked the required initiative in organizing information given within the multimodal sources.

Half of the included interventions were delivered to CYP with ASD, and much of the research to date evaluating digital technologies administered to NDDs has focused on ASD [41-43]. A possible explanation for this is that computer technology can help compensate verbal and social interaction difficulties and enable facilitation of exchanges between people with ASD, experts, and others [44]. The vast potential of technology for ASD has been realized by researchers, as technologies can enable new ways of communicating for people with ASD, socializing, and even learning. Despite this, many studies still lack scientific rigor to allow for concrete support for the use of technology in aiding people with ASD [42]. In this review, 2 of the 5 RCTs found that Web-based interventions were effective for CYP with ASD and one of these targeted CYP with HFASD who had a comorbid diagnosis of an anxiety disorder.

The RCTs included in this review were assessed as being of acceptable quality for a review of effectiveness. However, the main methodological issues centered on the lack of blinding of participants and of those delivering treatment. All studies had a control group, which was either active or inactive, with half of the trials using valid, standardized outcome measures. Most trials had low attrition rates thus improving the overall quality of the included studies. Only 1 of the 10 trials explicitly recorded and reported adverse events [29]. They reported on 10 adverse events that could be related to the intervention however, none were regarded as serious. Insufficient reporting of adverse events in psychological treatments has been documented in the literature [45], and it is clear that future trials should be more explicit in their reporting.

### Limitations

Some limitations of the review and meta-analysis need to be considered. A major limitation is the minimal number of studies retrieved meaning that any conclusions drawn from this review must be met with caution. To provide an expansive overview of the effectiveness of Web-based interventions for CYP, we included trials targeting a myriad of NDDs, which may have equilibrated disorder-specific effects of Web-based interventions. As there were very few RCTs evaluating the effectiveness of Web-based interventions in CYP with NDDs, it would have been impractical to carry out a review focusing on 1 NDD only. We could have increased the number of NDDs by also including trials focusing on CYP with learning disabilities; however, this would have further increased the heterogeneity and added to the problems of generalizability owing to the complexity of this particular population. The search was conducted on multiple databases and updated through a repeated search, thus ensuring a comprehensive overview of the topic. A particular strength of this review is that we had 2 independent reviewers screening relevant papers, with discrepancies between the reviewers discussed. This ensured a structured, meticulous approach was undertaken in study selection, therefore, improving review quality.

For the meta-analysis, we could only include data from 5 of the 10 trials, meaning the pool of data from included interventions was small and limited the overall power. Moreover, there was a high level of heterogeneity detected in the meta-analysis, which may have been because of the types of comparison with the interventions or differences in baseline symptomology [46]. There is mixed literature on whether a meta-analysis should be conducted at all in the event of high heterogeneity; however, experts recommend using the random effects model [21,47] that was used in this review. Finally, a major strength of this review is that it is based on *a priori* protocol that decreases the potential for reviewer bias.

When interpreting the findings, some inherent methodological issues of the included studies must also be considered, as methodological flaws of the primary trials can have a considerable impact on the review results. One intrinsic methodological limitation of many therapeutic intervention trials is the lack of blinding of participants and those delivering treatment [48], thus introducing a high risk of bias. As already mentioned, most of the included trials had very small sample sizes, which makes the generalization of findings highly problematic. All interventions used different content and modalities of delivery, which could have affected participant interaction and consequently, effectiveness [49]. Another limitation is with the RCT design itself. Given that the most effective interventions are individualized to the user [17], this is often difficult to assess using an RCT design, meaning the interventions reviewed mostly fell short on this dimension.

Gender balance was a potential issue of bias in included studies, as most of the trials had more male participants than female. However, this is not surprising given that NDDs are more common in males than females [50]. Baseline symptomology was also a potential source of bias, as this may have caused difficulties comparing intervention effectiveness in improving NDD outcomes. Some trials recruited participants with minimal symptoms, whereas others recruited those experiencing high levels of NDD symptoms. Despite these limitations, the overall reporting of the included trials was of a high standard and methodologically sound.

### Implications for Practice

As some of the interventions found positive outcomes, health care professionals working with CYP may want to consider utilizing Web-based and digital resources to support their patients, especially those with tics. The National Health Service (NHS) has already developed improving access to psychological therapy services for young people with mental health problems and is aiming to incorporate this into practice nationwide within the coming years [51]. If this is successful in reducing the burden on health care services and is shown to be cost-effective, this could lead to promising new developments for digital resources to be used on other populations. None of the included studies assessed the cost-effectiveness of Web-based interventions, which is likely to be an important consideration for policymakers. All the efficacious interventions in this review contained an element of human interaction, either with a real person by videoconferencing or a simulated person in a virtual environment or serious game. The best improvement in outcomes, therefore, may be achieved through a combination of Web-based interventions and human support. As technology evolves rapidly, future Web-based interventions will be more dynamic, perhaps including real-time clinician or therapist input and integrated synchronous crisis support. A promising new development is the use of virtual reality, which has had positive results on children with ADHD [52], adults with anxiety disorders [53], and a range of other mental health problems [54]. Developers could utilize virtual reality to its full effect and enable a simulated, life-like human therapist to support CYP with NDDs and common comorbidities, thus cutting waiting lists while improving outcomes.

### Implications for Research

Future studies of Web-based interventions for CYP with NDDs must have larger sample sizes to generate a reasonable degree of statistical power and allow for an increase in generalizability. They must also consider including long-term follow-up assessments to evaluate whether effects are maintained over a prolonged period. A cost-effectiveness evaluation would also be appropriate and much needed in future research. Furthermore, qualitative feedback in the form of a process evaluation would be useful in addressing the intervention’s mechanisms of impact and usability.

Our review found multiple methodological issues with the included trials. Sources of high risk of bias in the RCTs included failure to blind participants and personnel to the Web-based intervention and inadequate reporting of allocation concealment. Failing to blind participants, which can be difficult in Web-based intervention studies, can lead to the *digital placebo effect* [55]. One possible way of mediating this effect in future studies is to create a sham or static Web-based program for control groups, therefore, reducing the risk of the digital placebo effect. As mentioned, individualized interventions are often the most effective; however, RCT designs are inadequate in assessing the individualized dimension of interventions, therefore future studies should focus on conducting single case experimental designs to measure this [56,57].

### Conclusions

Technological advances and mobile device popularity have huge potential to improve outcomes in CYP with NDDs and comorbid psychological problems. Overall, this study suggests that Web-based interventions can be beneficial in improving symptoms in this population; however, because of the small number of RCTs yielded and several methodological limitations in the included studies, mean findings must be considered with caution. There need to be more studies with larger sample sizes assessing the effectiveness of Web-based interventions for CYP. Furthermore, a qualitative evaluation of the intervention is encouraged in future work to provide bespoke Web-based interventions for youth populations.

## Appendix

Multimedia Appendix 1 Full search strategy.

Multimedia Appendix 2 Further information.

## Abbreviations

ADHD: Attention deficit hyperactivity disorder
ASD: Autism Spectrum Disorder
CBIT: Comprehensive Behavioural Intervention for Tics
CBT: cognitive behavioral therapy
CG: control group
CTD: Chronic Tic Disorder
CYP: children and young people
DSM: Diagnostic and Statistical Manual of Mental Disorder
ER: emotion recognition
F2F: face-to-face
HFASD: high-functioning autism spectrum disorder
ITT: intention-to-treat
JBI: Joanna Briggs Institute
LD: learning disorder
MEDLINE: Medical Literature Analysis and Retrieval System Online
NDD: neurodevelopmental disorder
NHS: National Health Service
NIHR: National Institute for Health Research
OT: occupational therapy
RCT: randomized controlled trial
SMD: standardized mean difference
SUMARI: System for the Unified Management, Assessment and Review of Information
TAU: treatment as usual
TD: tic disorder
TOBY: Therapy Outcomes By You
VMI: visual-motor integration
WLC: waitlist control
