# Supplementary material for: The Effectiveness of Web-Based Interventions Delivered to Children and Young People With Neurodevelopmental Disorders: Systematic Review and Meta-Analysis
Source: J Med Internet Res. 2019 Nov 1;21(11):e13478. doi: 10.2196/13478 (PMC6858614; doi:10.2196/13478)
Supplement: Multimedia Appendix 1 [file jmir_v21i11e13478_app1.pdf]

Multimedia Appendix 1

Search strategy - OVID SP interface

1 neurodevelopmental disorders/

2 child behavior disorders/

3 developmental disorder/

4 exp attention deficit disorder/

5 hyperkinesia/

6 exp autism/

7 exp asperger syndrome/

8 exp tic/

9 motor skills disorders/

10 stereotypic movement disorder/

11 communication disorders/

12 childhood-onset fluency disorder/

13 social communication disorder/

14 speech sound disorder/

15 Specific Learning Disorder/

16 exp developmental language disorder/

17 Intellectual Disability/

18 learning disorder/ or dyscalculia/

19 "Neurodevelopmental disorder*".tw.

20 "Developmental disorder*".tw.

21 (attenti* adj2 (deficit* or disorder*)).tw.

22 (adhd or addh or "ad hd" or ad??hd).tw.

23 ((hyperkin* or "hyper kin*" or hyper-kin*) adj2 (syndrome* or disorder*)).tw.

24 "pervasive development* disorder*".tw.

25 (autistic or autism or asperger* or "Kanner* syndrome" or "childhood disintegrative disorder").mp. or Rett*.tw.

26 (Tourette* or "tic disorder*").tw.

27 ("Stereotyp* movement disorders" or "stammering" or "cluttering").tw.

28 ("Communication Disorder*" or "Language* Disorder*" or "specific language impairment").tw.

29 ("Speech Sound Disorder*" or "Childhood?Onset Fluency Disorder*" or Stuttering or "Speech articulation disorder*" or "phonological disorder*" or "specific developmental disorder* of speech and language" or "specific speech articulation disorder").tw.

30 "Global developmental delay".tw.

31 ((intellectual* or learning*) adj3 (impair* or disab* or disorder* or difficult*)).tw.

32 ("Specific Learning Disorder*" or "Specific reading disorder" or "Disorder of written expression" or "Mathematics disorder" or "specific spelling disorder" or "dyslexia" or "disorder of arithmetical skills" or "dyscalculia" or "Specific developmental disorder of motor function" or "dyspraxia" or "developmental co?ordination disorder").tw.

33 1 or 2 or 3 or 4 or 5 or 6 or 7 or 8 or 9 or 10 or 11 or 12 or 13 or 14 or 15 or 16 or 17 or 18 or 19 or 20 or 21 or 22 or 23 or 24 or 25 or 26 or 27 or 28 or 29 or 30 or 31 or 32

34 exp Mobile Applications/ or (Application or Applications or App or Apps or Intervention or Interventions).mp. or ((Smartphone or Smart-phone or Smart phone or Smartphones or Smart-phones or Smart phones or Mobile or iPhone or Android) adj2 (Application or Applications or App or Apps or Intervention or Interventions)).ti,ab. [mp=title, abstract, original title, name of substance word, subject heading word, floating sub-heading word, keyword heading word, protocol supplementary concept word, rare disease supplementary concept word, unique identifier, synonyms]

35 (Internet or computer or computer* or online or web or e-therapy or e-mental or e-health or telehealth or telecare or teletherapy or telemedicine or telemental or technolog* or virtual or cyber or cyberpsychology or cybertherapy or iCBT or cCBT or web-based or web-guided or web-supported or web-delivered or web-assisted or web-aided or web-facilitated or computer-based or computer-guided or computer-supported or computer-delivered or computer-assisted or computer-aided or computer-facilitated or internet-based or internet-guided or internet-supported or internet-delivered or internet-assisted or internet-aided or internet-facilitated or online-based or online-guided or online-supported or online-delivered or online-assisted or online-aided or online-facilitated).ti,ab.

36 34 or 35

37 (adolescence or adolescent or adolescent development or boy or child or childhood or elementary student or girl or high school student or high school or kindergarten or middle school student or middle school or preschool child or puberty or student or minors or adolescent psychiatry or adolescent psychology or adolescent psychotherapy or adolescent psychopathology or child psychotherapy or child psychiatry or child* or juvenile* or teen*).ti,ab.

38 33 and 36 and 37

39 limit 38 to (humans and yr="2000 -Current" and randomized controlled trial)

Web of Science

#5 AND #4

Indexes=SCI-EXPANDED, SSCI, A&HCI, CPCI-S, CPCI-SSH, BKCI-S, BKCI-SSH, ESCI, CCR-EXPANDED, IC Timespan=2000-2018

5

TS=(RCT or Randomised Control or Randomized Contro*)

Indexes=SCI-EXPANDED, SSCI, A&HCI, CPCI-S, CPCI-SSH, BKCI-S, BKCI-SSH, ESCI, CCR-EXPANDED, IC Timespan=2000-2018

4

#3 AND #2 AND #1

Indexes=SCI-EXPANDED, SSCI, A&HCI, CPCI-S, CPCI-SSH, BKCI-S, BKCI-SSH, ESCI, CCR-EXPANDED, IC Timespan=2000-2018

3

TS=(adolescence or adolescent or boy or child or childhood or elementary student or girl or high school student or high school or kindergarten or middle school student or middle school or preschool child or puberty or student or minors or child* or juvenile* or teen*)

Indexes=SCI-EXPANDED, SSCI, A&HCI, CPCI-S, CPCI-SSH, BKCI-S, BKCI-SSH, ESCI, CCR-EXPANDED, IC Timespan=2000-2018

2

TS=(neurodevelopmental disorders or Communication Disorders or Language Disorder or Speech Sound Disorder or Childhood-Onset Fluency Disorder or Stuttering or Social Pragmatic Communication Disorder or Unspecified Communication Disorder or Autism Spectrum Disorder or Autism Spectrum Disorder or Attention-Deficit/Hyperactivity Disorder or Attention-Deficit/Hyperactivity Disorder or Other Specified Attention-Deficit/Hyperactivity Disorder or Unspecified Attention-Deficit/Hyperactivity Disorder or Specific Learning Disorder or Specific Learning Disorder or Motor Disorders or Developmental Coordination Disorder or Stereotypic Movement Disorder or Tic Disorders or Other Specified Tic Disorder or Unspecified Tic Disorder or Other Neurodevelopmental Disorders or Other Specified Neurodevelopmental Disorder or Unspecified Neurodevelopmental Disorder)

Indexes=SCI-EXPANDED, SSCI, A&HCI, CPCI-S, CPCI-SSH, BKCI-S, BKCI-SSH, ESCI, CCR-EXPANDED, IC Timespan=2000-2018

1

TS=(Mobile Applications or Application or Applications or App or Apps or Intervention or Interventions or Smartphone or Smart-phone or Smart phone or Smartphones or Smart-phones or Smart phones or Mobile or iPhone or Android or Application or Applications or App or Apps or Intervention or Interventions or Internet or computer or computer* or online or web or e-therapy or e-mental or e-health or telehealth or telecare or teletherapy or telemedicine or telemental or technolog* or virtual or cyber or cyberpsychology or cybertherapy or iCBT or cCBT or web-based or web-guided or web-supported or web-delivered or web-assisted or web-aided or web-facilitated or computer-based or computer-guided or computer-supported or computer-delivered or computer-assisted or computer-aided or computer-facilitated or internet-based or internet-guided or internet-supported or internet-delivered or internet-assisted or internet-aided or internet-facilitated or online-based or online-guided or online-supported or online-delivered or online-assisted or online-aided or online-facilitated or

Therapy, Computer-Assisted or *Therapy, Computer-Assisted or Computer-assisted treatment or Web-based treatment or *Multimedia or Software or *Computer Simulation or Computerized intervention)

PubMed

(Communication Disorders or Language Disorder or Speech Sound Disorder or Childhood Onset Fluency Disorder or Stuttering or Social Pragmatic Communication Disorder or Unspecified Communication Disorder or Autism Spectrum Disorder or Autism Spectrum Disorder or Attention Deficit Hyperactivity Disorder or Attention Deficit Hyperactivity Disorder or Other Specified Attention Deficit Hyperactivity Disorder or Unspecified Attention Deficit Hyperactivity Disorder or Specific Learning Disorder or Specific Learning Disorder or Motor Disorders or Developmental Coordination Disorder or Stereotypic Movement Disorder or Tic Disorders or Other Specified Tic Disorder or Unspecified Tic Disorder or Other Neurodevelopmental Disorders or Other Specified Neurodevelopmental Disorder or Unspecified Neurodevelopmental Disorder) in Title Abstract Keyword

AND

(Mobile Applications or Application or Applications or App or Apps or Intervention or Interventions or Smartphone or Smart-phone or Smart phone or Smartphones or Smart-phones or Smart phones or Mobile or iPhone or Android or Application or Applications or App or Apps or Intervention or Interventions or Internet or computer or computer* or online or web or e-therapy or e-mental or e-health or telehealth or telecare or teletherapy or telemedicine or telemental or technolog* or virtual or cyber or cyberpsychology or cybertherapy or iCBT or cCBT or web-based or web-guided or web-supported or web-delivered or web-assisted or web-aided or web-facilitated or computer-based or computer-guided or computer-supported or computer-delivered or computer-assisted or computer-aided or computer-facilitated or internet-based or internet-guided or internet-supported or internet-delivered or internet-assisted or internet-aided or internet-facilitated or online-based or online-guided or online-supported or online-delivered or online-assisted or online-aided or online-facilitated or Therapy, Computer-Assisted or *Therapy, Computer-Assisted or Computer-assisted treatment or Web-based treatment or *Multimedia or Software or *Computer Simulation or Computerized intervention) in Title Abstract Keyword

AND

(adolescence or adolescent or boy or child or childhood or elementary student or girl or high school student or high school or kindergarten or middle school student or middle school or preschool child or puberty or student or minors or child* or juvenile* or teen*) in Title Abstract Keyword - (Word variations have been searched)

Central

(Communication Disorders or Language Disorder or Speech Sound Disorder or Childhood Onset Fluency Disorder or Stuttering or Social Pragmatic Communication Disorder or Unspecified Communication Disorder or Autism Spectrum Disorder or Autism Spectrum Disorder or Attention Deficit Hyperactivity Disorder or Attention Deficit Hyperactivity Disorder or Other Specified Attention Deficit Hyperactivity Disorder or Unspecified Attention Deficit Hyperactivity Disorder or Specific Learning Disorder or Specific Learning Disorder or Motor Disorders or Developmental Coordination Disorder or Stereotypic Movement Disorder or Tic Disorders or Other Specified Tic Disorder or Unspecified Tic

Disorder or Other Neurodevelopmental Disorders or Other Specified Neurodevelopmental Disorder or Unspecified Neurodevelopmental Disorder) in Title Abstract Keyword

AND

(Mobile Applications or Application or Applications or App or Apps or Intervention or Interventions or Smartphone or Smart-phone or Smart phone or Smartphones or Smart-phones or Smart phones or Mobile or iPhone or Android or Application or Applications or App or Apps or Intervention or Interventions or Internet or computer or computer* or online or web or e-therapy or e-mental or e-health or telehealth or telecare or teletherapy or telemedicine or telemental or technolog* or virtual or cyber or cyberpsychology or cybertherapy or iCBT or cCBT or web-based or web-guided or web-supported or web-delivered or web-assisted or web-aided or web-facilitated or computer-based or computer-guided or computer-supported or computer-delivered or computer-assisted or computer-aided or computer-facilitated or internet-based or internet-guided or internet-supported or internet-delivered or internet-assisted or internet-aided or internet-facilitated or online-based or online-guided or online-supported or online-delivered or online-assisted or online-aided or online-facilitated or Therapy, Computer-Assisted or *Therapy, Computer-Assisted or Computer-assisted treatment or Web-based treatment or *Multimedia or Software or *Computer Simulation or Computerized intervention) in Title Abstract Keyword

AND

(adolescence or adolescent or boy or child or childhood or elementary student or girl or high school student or high school or kindergarten or middle school student or middle school or preschool child or puberty or student or minors or child* or juvenile* or teen*) in Title Abstract Keyword - (Word variations have been searched)

Clinicaltrials.gov

Completed Studies | Interventional Studies | Neurodevelopmental Disorders | online therapy | Child
