# Supplementary material for: The Effectiveness of Web-Based Interventions Delivered to Children and Young People With Neurodevelopmental Disorders: Systematic Review and Meta-Analysis
Source: J Med Internet Res. 2019 Nov 1;21(11):e13478. doi: 10.2196/13478 (PMC6858614; doi:10.2196/13478)
Supplement: Multimedia Appendix 2 [file jmir_v21i11e13478_app2.pdf]

Multimedia Appendix 2

Assessment of methodological quality

This tool includes 13 questions, which aid in our understanding of trial quality by assessing study bias across the following domains: random sequence generation, allocation concealment, blinding, incomplete follow-up data, selective reporting, the reliability of outcomes measures, appropriateness of statistical analysis utilized, and appropriateness of trial design to the particular study. Blinding is further divided into 'blinding of participants', 'blinding of those delivering treatment' and 'blinding of outcome assessors.' Criteria were scored as being 'met', 'not met', 'unclear' or 'not applicable' by the two assessors independently, with any disagreements being discussed or, where necessary, a third assessor was consulted. By using the JBI RCT appraisal tool instead of the Cochrane Risk of Bias Tool was a deviation from the protocol for this review. The reason for this deviation was that the JBI RCT appraisal tool is more comprehensive and for reasons of consistency in using JBI methodology throughout this review.

Adverse events and outcome measures

All studies administered outcome measures at baseline and post-treatment, one study [26], also had a mid-point follow-up and four studies had follow-up points at three [22], four [27], five [29], and six [24] months. A variety of measures was employed to assess outcomes. The majority of trials administered a combination of clinician, self and parent report measures — one study [29] utilized a teacher report measure as well — which were administered either through hard copies or through online access. In all but two studies, measures used were standardized measurements, except one study [31], which employed a standardized arithmetic test specific to Brazilian schools, and another study [23], which gave children specific targets to achieve within the app.

Response burden refers to the extent to which participants are strained by completing measures, such as the length and intensity of the outcome measure. RCTs, in particular, must consider this, as participants typically complete measures at multiple time-points, which may result in large attrition rates. We calculated the number of items participants

completed through totaling the approximated number of items within administered measures in the included studies.

Secondary outcomes

Secondary outcomes of significance were comorbid psychological symptoms targeted and only one of the ten studies aimed to reduce this. In the study by Conaughton et al. [22], children with HFASD and comorbid anxiety in the completer sample were free of their primary anxiety diagnosis at post-treatment compared to the WLC group (20% vs 0%), with 38.9% of the intervention group being free of their primary anxiety diagnosis at 3-month follow-up. With respect to loss of all anxiety diagnoses, 10% of the intervention group versus 0% of the WLC group had lost all anxiety diagnoses at post-assessment, with 16.7% of the intervention group being free of all anxiety diagnoses at 3-month follow-up.

For the ITT sample, a higher percentage of participants in the intervention group (19%) were free of their primary anxiety diagnosis at post-assessment compared to the WLC group (0%), with 33.3% of the intervention group being free of their primary anxiety diagnosis at 3-month follow-up. With respect to loss of all anxiety diagnoses (for the ITT sample), 9.5% of the intervention group versus 0% of the WLC group had lost all anxiety diagnoses at post-treatment, with 14.3% of the intervention group being free of all anxiety diagnoses at 3-month follow-up.
